# Genome-wide association study and transcriptome of olecranon-type traits in peach (*Prunus persica L.*) germplasm

**DOI:** 10.1186/s12864-021-08017-y

**Published:** 2021-09-28

**Authors:** Jianliang Liu, Yao Bao, Yuming Zhong, Qin Wang, Huifan Liu

**Affiliations:** 1 College of Light Industry and Food, Zhongkai University of Agriculture and Engineering, 510225 Guangzhou, Guangdong China; 2 Guangzhou Key Laboratory for Research and Development of Crop Germplasm Resources, Zhongkai University of Agriculture and Engineering, 510225 Guangzhou, China; 3 Modern Agriculture Research Center, Zhongkai University of Agriculture and Engineering, 510225 Guangzhou, Guangdong China; 4 Guangdong Provincial Key Laboratory of Lingnan Specialty Food Science and Technology, Guangzhou, Guangdong China; 5 College of Environmental Science and Engineering, Zhongkai University of Agriculture and Engineering, 510225 Guangzhou, Guangdong China

**Keywords:** Olecranon honey peach, Full-length transcriptome sequencing, Genome-wide association study

## Abstract

**Background:**

The top of the olecranon honey peach (*Prunus persica L.*) fruit appears similar to an eagle’s beak. In this study, a single olecranon honey peach with a round-type fruit was observed in our fruit orchard. To explore the genetic mechanism of olecranon formation, we performed full-length transcriptome sequencing analysis of olecranon and round peaches as well as a genome-wide association study of the association of olecranon-type trait loci.

**Results:**

The gene locus was 26,924,482 base pairs in NC_034014.1. Transcriptome sequencing showed that the clean sequencing data of each sample reached 7.10GB, with 14,360 genes and 23,167 transcripts expressed in both the olecranon honey peach and round peach. Among the 11 differentially expressed genes selected as candidate genes, six were highly expressed in olecranon peach and named as *LOC18775282*, *LOC18772209*, *LOC18773929*, *LOC18772013*, *LOC18773401*, and *ONT.13798.5*. Five genes were highly expressed in round peach and named as *LOC18773079*, *LOC18773525*, *LOC18773067*, *LOC18775244*, and *LOC18772236*. Notably, *ONT.13798.5* was not previously identified. The genes were within 1 Mb up- or down-stream of the main genome-wide association study locus for olecranon-type traits.

**Conclusions:**

This study revealed loci associated with olecranon and provides useful information for analysis and breeding of olecranon honey peach.

**Supplementary Information:**

The online version contains supplementary material available at 10.1186/s12864-021-08017-y.

## Background

Peach (*Prunus persica L.*) is a genetic and genomic reference species for the genus *Prunus* because of its small genome and relatively short juvenile period [1]. Cultivated peach has a diploid (2n = 2x = 16) and relatively small genome (265 Mb) (www.rosaceae.org*).* Peach shape is an important external characteristic used by consumers to select preferred cultivars [2]. The shape of peach can be classified as round or flat; flat peaches are more popular than round peaches [2, 3]. Most studies of peach shape have focused on flat and round peaches. However, few studies have examined the olecranon honey peach, which was named for its unique surface features, as the top resembles the beak of an eagle. It is considered as one of the best peach varieties and is very popular in China because it is sweet, juicy, and has a crisp texture [4].

Genome-wide association studies (GWAS) were used to identify flat- and round-related loci or genes, indicating that *PpCAD1* (*ppa003772m*, designated as *PpCAD1*) and 25,060,196 base pairs (bp) of scaffold Pp06 play important roles in the fruit shape [5]. The flat shape is determined by a single gene, S/s, in the distal part of chromosome 6, and the flat phenotype is caused by the partial dominant allele in heterozygosis, from which the dominant homozygote is aborted [5, 6]. Moreover, at 26,924,482 bp of scaffold Pp06, all accessions with round-shaped fruit were found to have an A/A genotype, whereas those with A/T or T/T genotypes formed flat-shaped fruits [3]. In addition, differences in the fruit cell number, cell size, and gene expression patterns between the round and flat fruit varieties and the oblate shape of peach fruits are mainly determined by regulating the production of cells in the vertical direction during early fruit development [2]. However, the molecular features affecting the shape of olecranon honey peach have not been evaluated in genetic or transcriptome studies.

In this study, a GWAS of olecranon traits in 456 peach accessions from across China was conducted to identify gene loci related to fruit shape. The Oxford Nanopore Technologies (ONT, Oxford, UK) sequencing method was used to study differences in the transcription of genes affecting the olecranon trait to narrow the range of related genes and provide a basis for further studies of these genes.

## Results

### Resequencing 456 accessions of peach

**Fig. 1:**
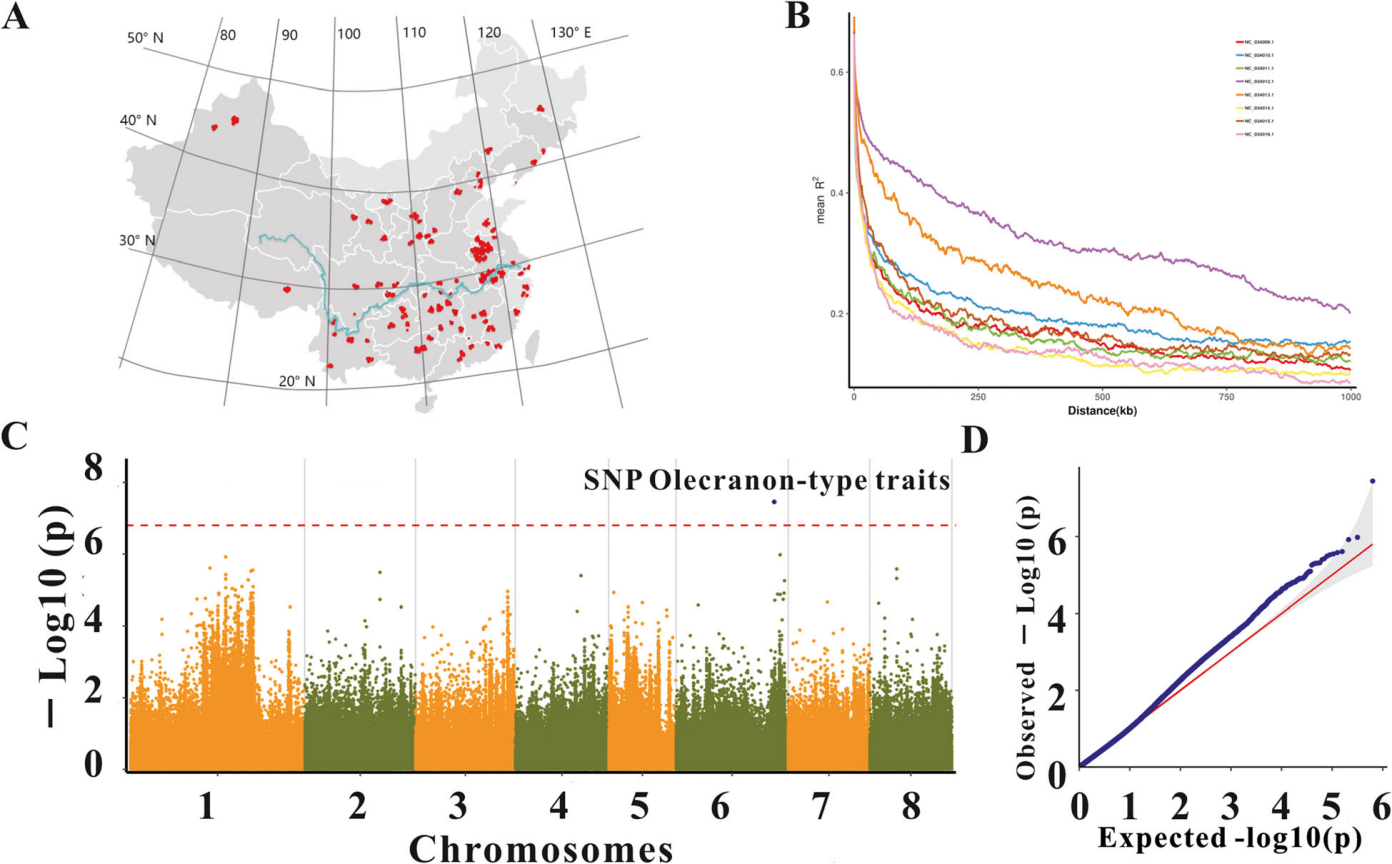
Geographic distribution, linkage disequilibrium (LD) decay of 456 peach accessions and genome-wide association scan for olecranon-type traits. (**A**) Geographic distribution of 456 peach accessions. (**B**) LD decays of each chromosome. (**C**) Manhattan plots for FaST-LMM. Red horizontal lines indicate genome-wide significance. (**D**) Quantile–quantile plot for FaST-LMM. The horizontal axis shows − log10-transformed expected *P*-values and vertical axis shows − log10-transformed observed *P*-values

We generated 2814.05Gbp clean reads, and the Q30 reached 92.43 % in whole-genome resequencing of 456 peach accessions from China (Fig. 1 A, Supplemental Table 1) with an average depth of 20X and coverage of 98.76 % for each accession (Supplemental Table 2). The average number of clean reads of the samples was 41,190,056, and the average mapping rate between the samples and reference gene was 97.38 % (Supplemental Table 2). The reads of each accession were mapped against the peach reference genome (release version NCBIv2), and a final set of 2,923,674 high-quality single-nucleotide polymorphisms (SNPs) and 627,795 insertions-deletions (InDels) was identified using SnpEff software. Averages of 231,407 and 121,336 SNPs were the transition type and transversion type, respectively, and the average SNP ratio of the heterozygous types was 63.39 % (Supplemental Table 3). For each accession, we analyzed the distribution of polymorphisms in each genomic region and found that averages of 96,783, 51,340, 85,430, and 63,805 SNPs were within intergenic regions, introns, upstream regions (within 5 kb upstream of transcription start sites), and downstream regions (within 5 kb downstream of transcription stop sites), respectively (Supplemental Table 4). In coding regions, averages of 16,305 nonsynonymous, 25 start-lost, 290 stop-gain, and 158 stop-lost SNPs were annotated (Supplemental Table 4), which led to amino acid changes, splicing variants, longer transcripts, or premature stop codons. Moreover, averages of 14,712, 11,377, 17,855, and 13,285 InDels were within intergenic regions, introns, upstream regions, and downstream regions, respectively (Supplemental Table 5).

### Patterns of LD across peach genome

Linkage disequilibrium (LD) analysis indicated that the physical distance between SNPs (reported as half of the maximum value) occurred at ~ 53.76 kb (r^2^ = 0.33) for *Amygdalus persica L.* (Table 1), which was slightly higher than that of wild peach (~ 35 kb) [7] and consistent with the narrow domestication bottleneck. The magnitude of LD decay varied among different chromosomes (Fig. 1D; Table 1). The maximum physical distance between SNPs was observed at ~ 292.11 kb (r^2^ = 0.35) for NC_034012.1, followed by NC_034013.1 (~ 132.42 kb). The minimum physical distance between SNPs occurred at ~ 20.84 kb (r^2^ = 0.34).

**Table 1:** LD blocks were defined using PLINK software

Chrom	r^2^ = (half_maxR^2^)	LD Decay (kb; r^2^ = half_maxR^2^)	LD Decay (kb; r^2^ = 0.1)
AmygdaluspersicaL	0.326383158	53.75822146	NA
NC_034009.1	0.318505145	27.89848112	NA
NC_034010.1	0.302647582	50.3113611	NA
NC_034011.1	0.339201279	20.84075695	NA
NC_034012.1	0.34596759	292.114469	NA
NC_034013.1	0.345084309	132.4155087	NA
NC_034014.1	0.315842494	25.85917109	853.7472139
NC_034015.1	0.334144895	38.01036343	NA
NC_034016.1	0.331580637	25.50060356	838.2343946

### GWAS of olecranon-type traits and prediction of candidate genes

In this study, we counted 296 types of peaches out of the 456 germplasm resources, among which 155 had olecranon and 141 had no olecranon. The peaches could also be divided into 82 nectarines and 225 peaches. The weights of these peaches ranged from 7.08 to 520 g, and the fruit shape index ranged from 0.60 to 1.33 (Supplemental Table 1). To explore the potential of GWAS to identify causal genes of olecranon-type traits, we used the FaST-LMM model to conduct an association study of olecranon-type traits. The locus defined by the SNP at 26,924,482 bp was strongly associated with olecranon-type traits (Fig. 1 C and 1D) and positioned on NC_034014.1, and the minor allele frequency was 0.07, which is greater than 0.05. The SNP locus 26,924,482 bp is homozygous in olecranon peach and heterozygous in round peach.

We selected 38 genes within 1 Mb up- or down-stream of the main GWAS locus for olecranon-type traits (Supplemental Table 6). Through Swiss-PROt database annotation, we found that 20 genes are also expressed in the *Arabidopsis* genome. Five genes were related to serine/threonine-protein kinase and six were related to non-specific lipid-transfer protein. This variation was highly associated with an A/T polymorphism detected in the fifth intron of *LOC18772384* on NC_034014.1. *LOC18772384* was annotated as MACPF domain-containing protein CAD1-like (Supplemental Table 6).

### Transcriptome material analysis

**Fig. 2:**
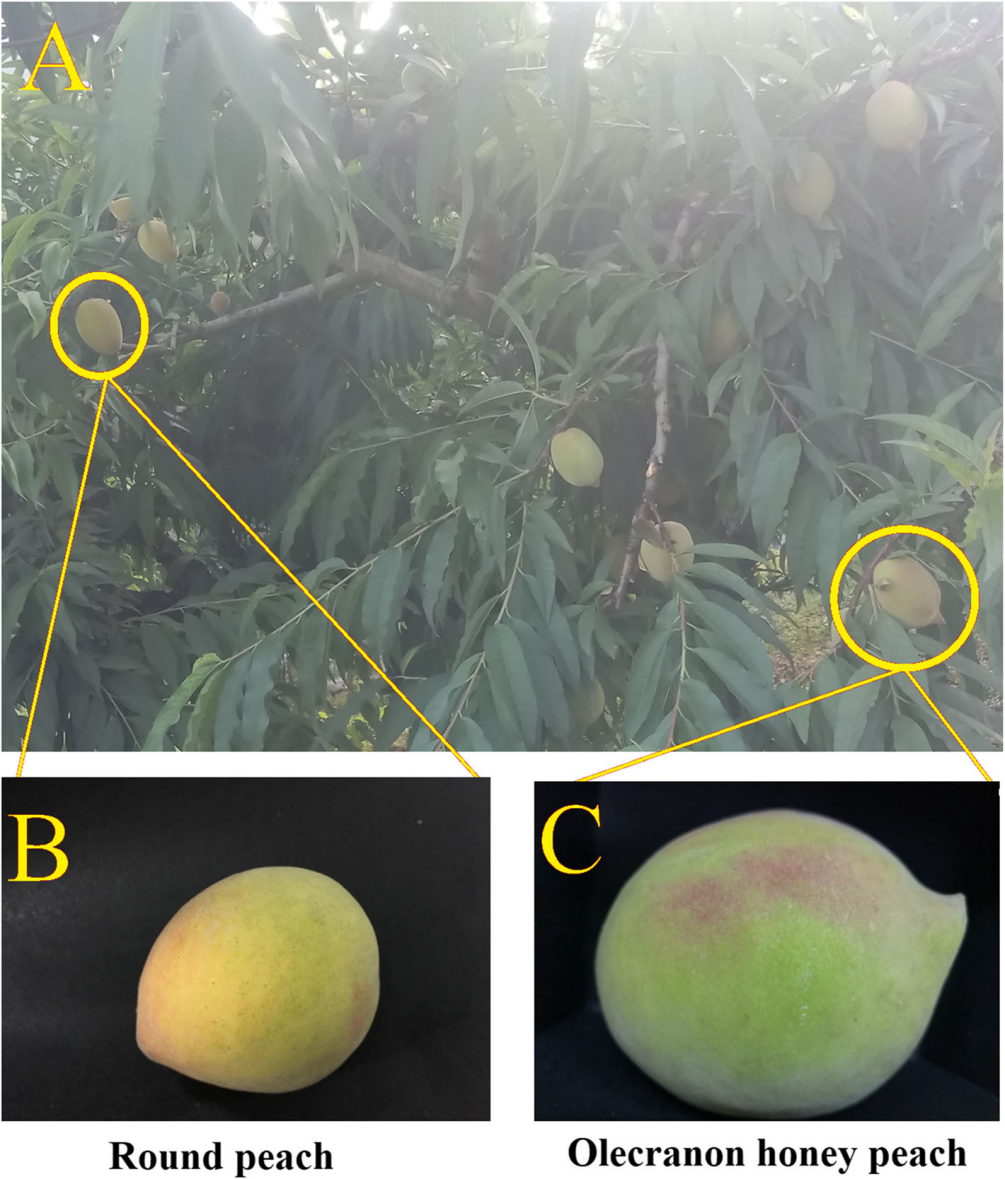
Phenotypic characterization of olecranon honey peach and round peach. (**A**) Sampled peach trees, (**B**) round peach, and (**C**) olecranon honey peach

A single olecranon honey peach tree with round-type fruits was observed in our fruit orchard. A branch of this tree was found to bear only round-type fruits (Fig. 2B), and was found to have an alteration related to fruit-shape in the bud sport. Moreover, other branches bore olecranon-type fruits (Fig. 2 C). To further distinguish fruits with round and olecranon shapes, ripe fruits were characterized (Table 2). The results showed that the olecranon-type fruit weighed 218.50 ± 4.36 g, and had a longitudinal diameter of 69.16 ± 0.49 cm and transverse diameter of 72.87 ± 1.10 cm; the round-type peach weighed 113.92 ± 1.72 g, and had a longitudinal diameter of 62.86 ± 0.63 cm and transverse diameter of 58.71 ± 0.94 cm. According to the *t*-test, the quality indicators of the two types of peaches differed significantly (*P* < 0.05). Notably, both the weight and vertical and horizontal diameters of olecranon-type fruit were larger than those of round-type fruit. However, the olecranon-type fruit shape index was smaller than that of round-type fruit; these values were 0.95 ± 0.01 and 1.07 ± 0.00, respectively.

**Table 2:** Comparison of quality indices of the two peach fruits

Project	Olecranon-type fruits	Round-type fruits
Weight (g)	218.49 ± 4.36	113.92 ± 1.72
Longitudinal diameter (cm)	69.17 ± 0.49	62.86 ± 0.63
Transverse diameter (cm)	72.87 ± 1.10	58.71 ± 0.94
Fruit shape index	0.95 ± 0.01	1.07 ± 0.00
SNP locus 26,924,482 bp	Homozygous	Heterozygous

### Overview of RNA-Seq data

The full-length transcriptome of six samples was sequenced, and the clean data of each sequence reached 7.10GB. After quality filtering, the read number ranged from 5,687,141 to 7,035,534 bp with an N50 ranging from 1263 to 1423 bp; the mean length of reads ranged from 1,145 to 1,259 bp, with a maximum length of 21,540 among all reads, and the mean q-score was Q9 in each library (Supplemental Table 7). After removing ribosomal RNA, the numbers of full-length reads in the six libraries were 5,195,328 (77.57 %), 481,897 (77.81 %), 4,795,588 (77.20 %), 5,346,222 (77.86 %), 4,312,262 (77.68 %), and 4,847,747 (76.97 %), respectively (Supplemental Table 8). The minimum number of reads per library was 5.55 million for B-2 and the maximum number was 6.87 million for B-1. Approximate 99.75 % of reads were mapped to the peach reference genome V2 (Supplemental Table 8). We analyzed the correlation between expression levels in different samples by Pearson correlation coefficient. The results for B-3 sample were abnormal (Supplementary Figure S1) and thus excluded from subsequent analysis.

### DEG and DET analysis

**Fig. 3:**
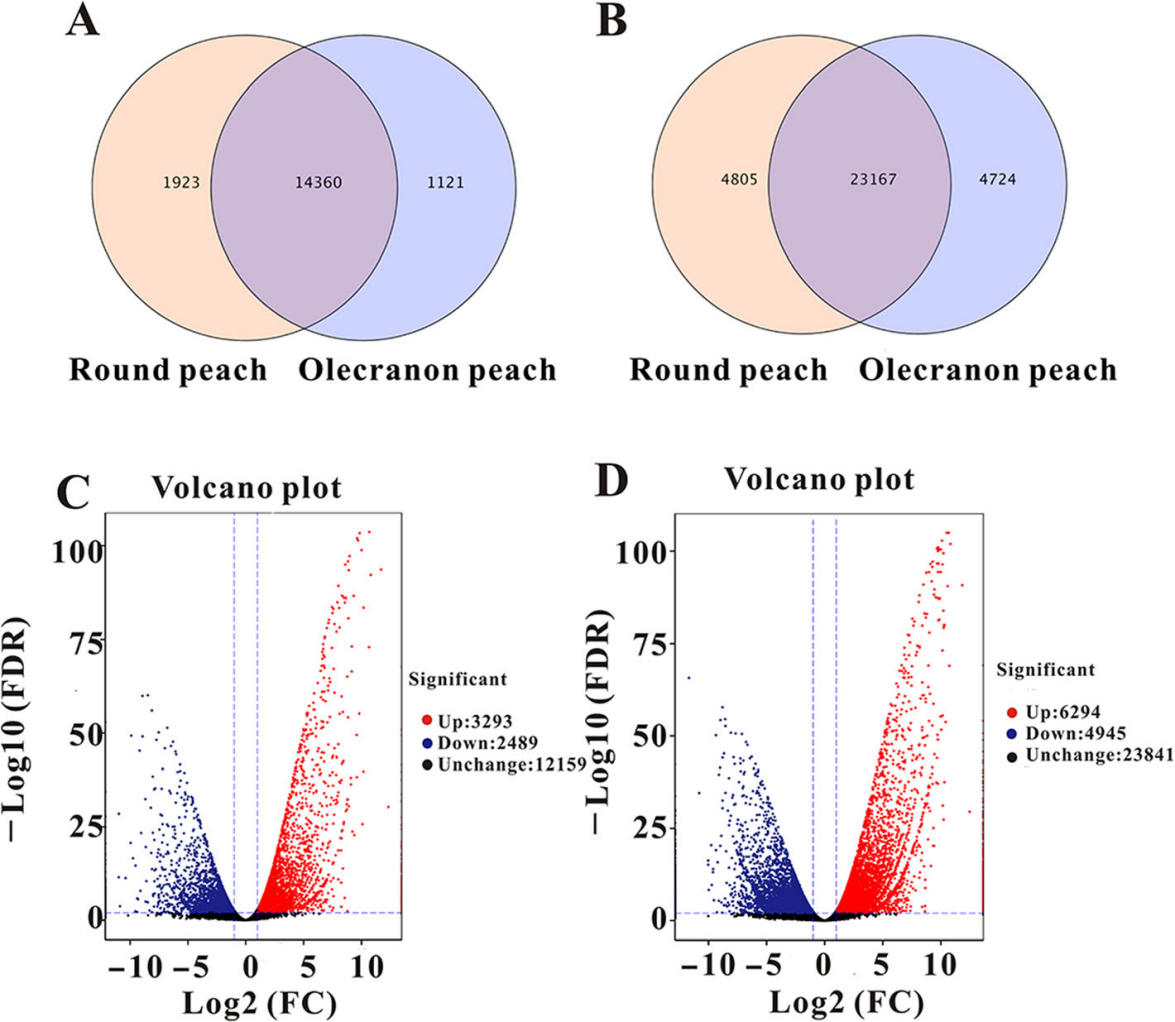
Expression of genes and transcriptome. (**A**) Venn diagram for genes in round peach and olecranon peach. (**B**) Venn diagram for transcriptome. (**C**) Volcano map of the differentially expressed genes (DEGs). Each point in the differential expression volcano chart represents a gene, and the abscissa represents the logarithm of the multiple of the difference in expression of a certain gene in the two samples; the ordinate represents the negative logarithm of statistical significance of the change in gene expression. A larger absolute value of the abscissa indicates a greater fold-difference in expression between the two samples; a larger ordinate value indicates more significant differential expression and greater reliability of the DEGs obtained by screening. The green dots in the figure represent down-regulated DEGs, red dots represent up-regulated DEGs, and black dots represent non-DEGs. (**D**) Volcano map of DEGs

We found 14,360 genes and 23,167 transcripts expressed in both olecranon and round peach (Fig. 3 A, 3B). There were 5782 differentially expressed genes (DEGs) in the two groups, including 3293 up-regulated genes and 2489 down-regulated genes with P-values < 0.05 (Fig. 3 C). There were 11,239 differentially expressed transcripts (DETs) in the two groups, including 6294 up-regulated transcripts and 4945 down-regulated transcripts with *P*-values < 0.05 (Fig. 3D).

### Functional annotation of DEGs and DETs

**Fig. 4:**
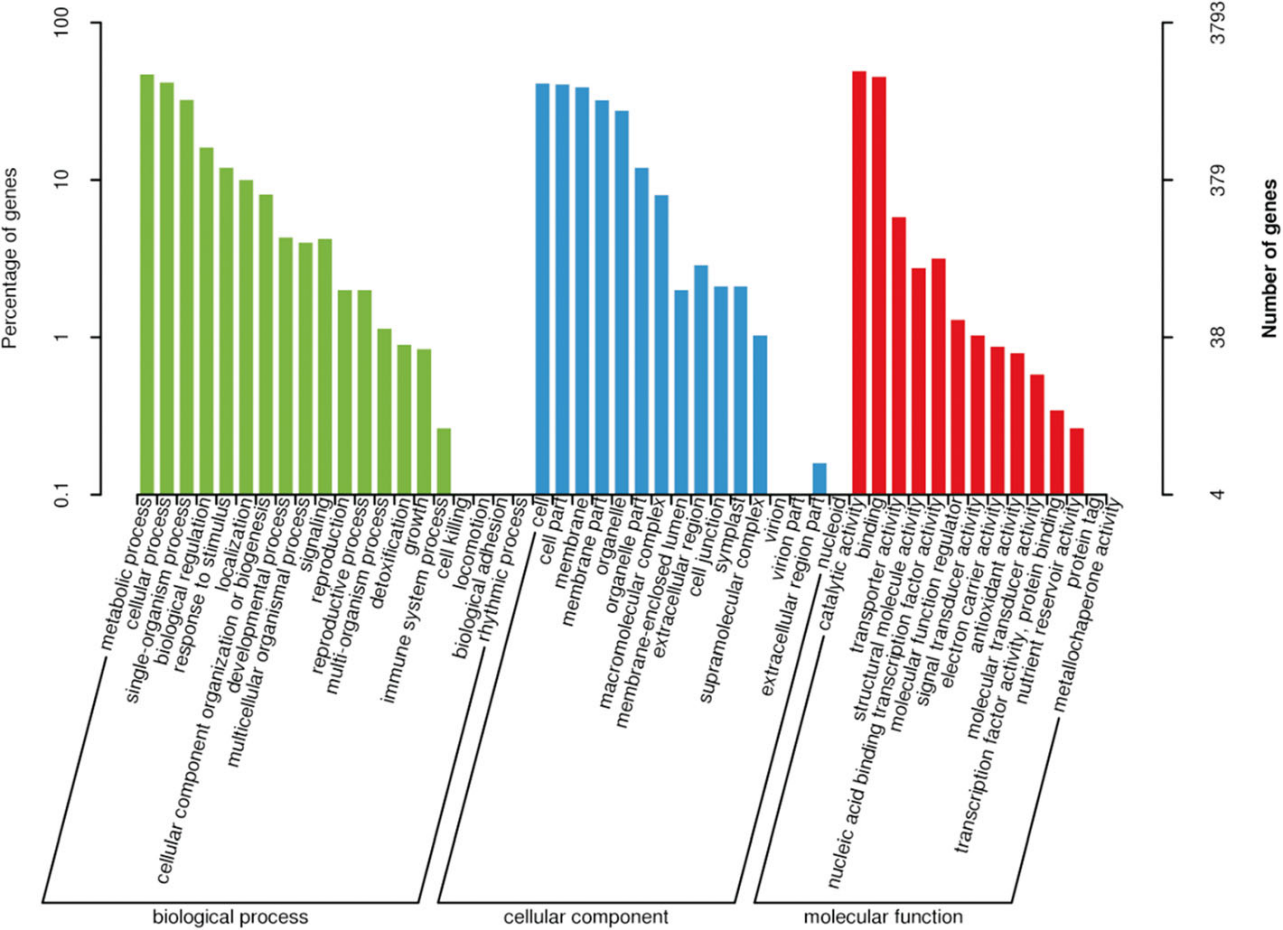
Statistical classification of Gene Ontology (GO) annotation for the differentially expressed genes. The abscissa represents the GO classification, left of the ordinate represents the percentage of the number of genes, and right represents the number of genes

**Fig. 5:**
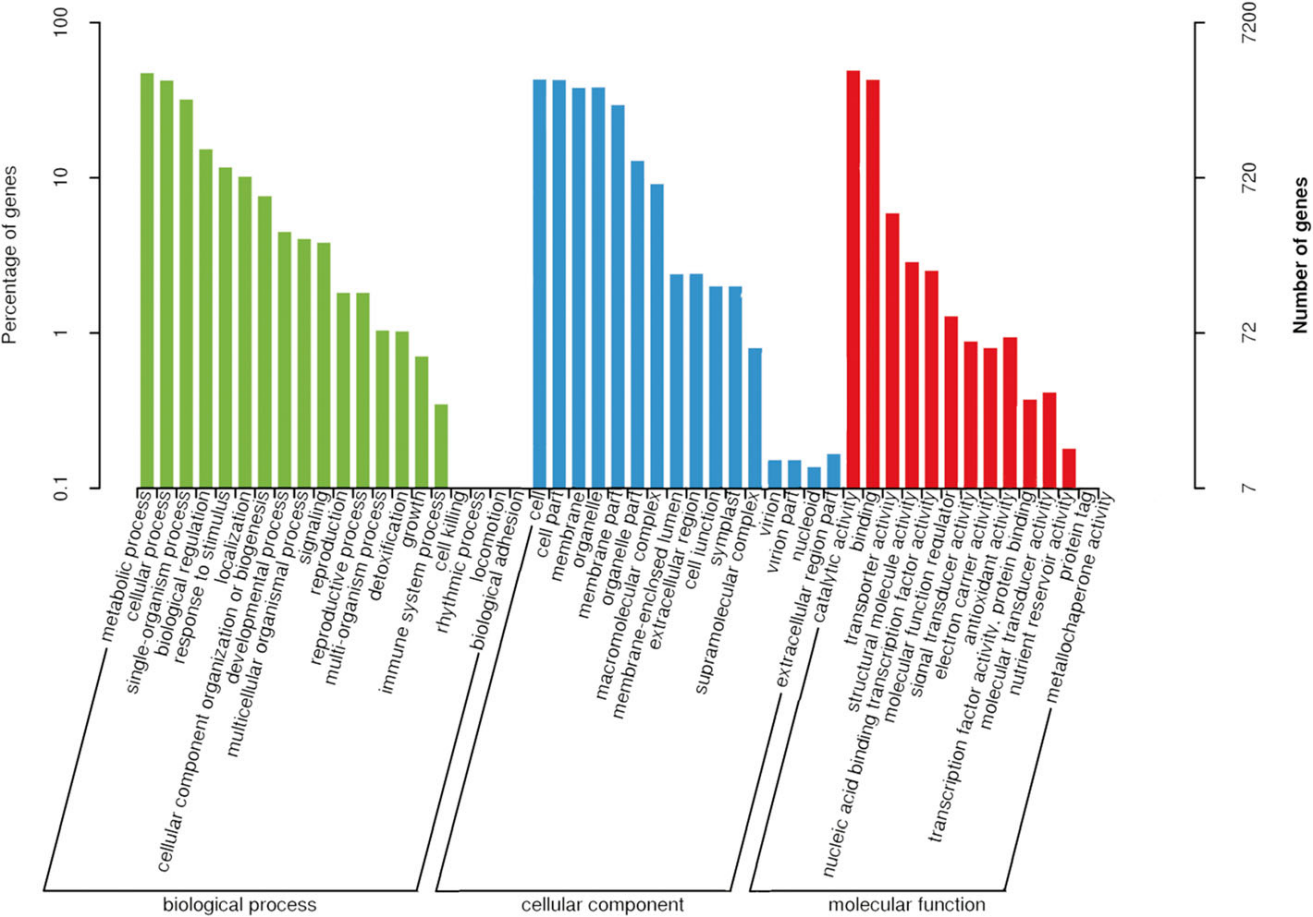
Statistical classification of Gene Ontology annotation for the differentially expressed transcripts

To identify genes important for olecranon-type traits, we annotated the DEGs and DETs using the Gene Ontology (GO), Kyoto Encyclopedia of Genes and Genomes (KEGG), Eukaryotic Orthologous Groups (KOG), Clusters of Orthologous Groups (COG), and eggNOG databases. Based on GO annotation, 3793 DEGs and 7200 DETs were classified into three main categories, including biological process, cellular component, and molecular function (Supplemental Tables 9, Figs. 4 and 5), with each category possessing 20, 16, and 14 terms, respectively. In the biological process classification, genes and transcripts related to metabolic process, cellular, and process were highly enriched. In the cellular component category, the main subcategories were cell and cell part. Catalytic activity and binding were the major subcategories in the biological process classification. A total of 1073 DEGs and 2293 DETs were enriched according to the KEGG database (Supplemental Table 9). The DEGs and DETs were divided into 5 categories, including cellular processes, environmental information processing, genetic information processing, metabolism, and organismal systems (Supplementary Figure S2 and S3). Among the DEGs, the most common term was plant hormone signal transduction related to environmental information processing. Among the DETs, the most common term was biosynthesis of amino acids related to metabolism. There were 3154 DEGs and 6222 DETs annotated in the KOG database (Supplementary Table 9). DEGs and DETs were most enriched in the eggNOG database, showing numbers of 5299 and 10,140, respectively (Supplementary Table 9).

### Selection of candidate genes for olecranon-type traits

**Fig. 6:**
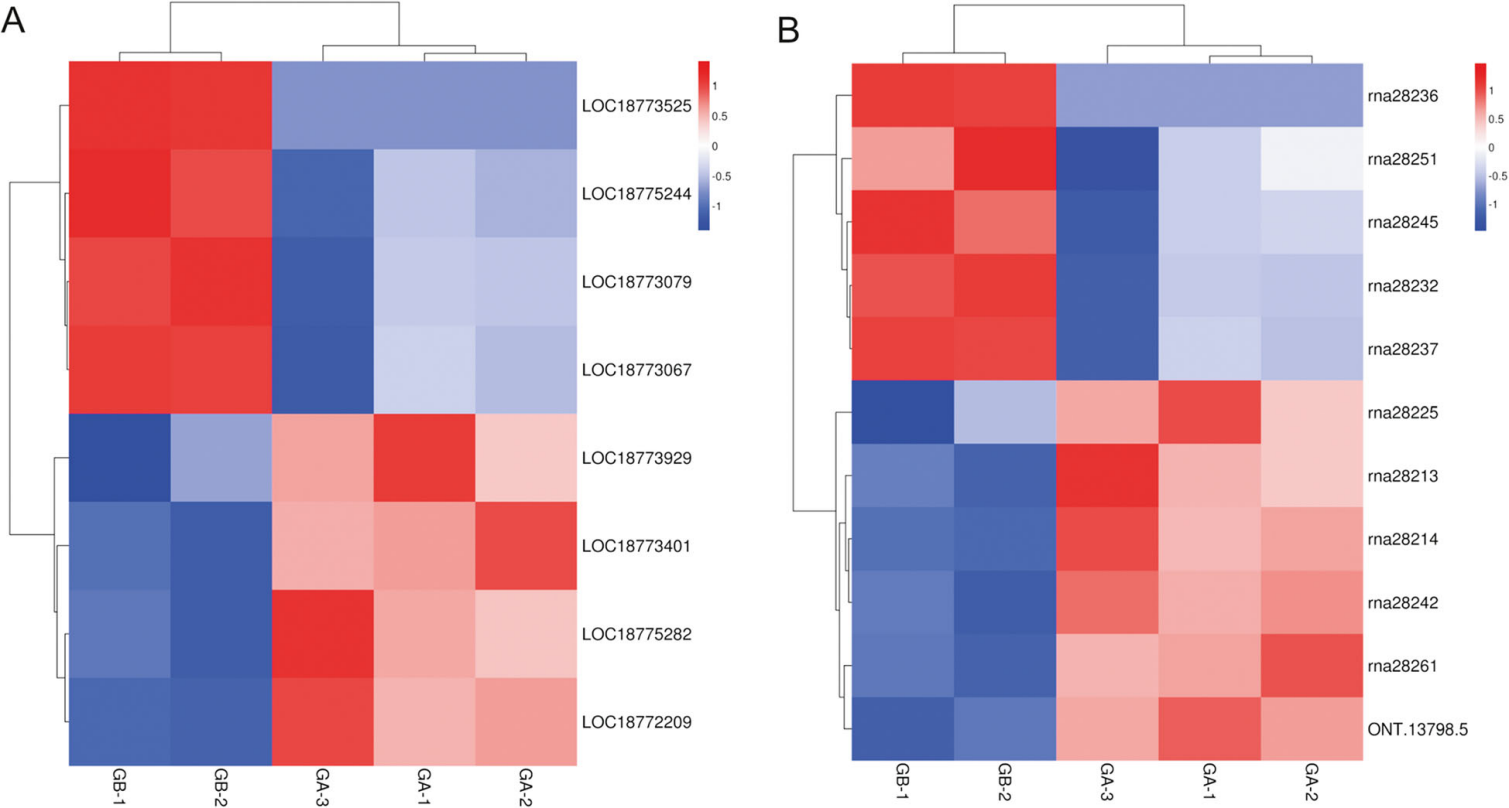
Expression level heatmap of selected differentially expressed genes (DEGs) and differentially expressed transcripts (DETs). (**A**) DEGs within 1 Mb up- or down-stream of 26,924,482 bp. (**B**) DETs within 1 Mb up- or down-stream of 26,924,482 bp. A-1; A-2; A-3: Olecranon honey peach; B-1; B-2; B-3: round peach

First, we selected 38 genes within 1 Mb up- or down-stream of the main GWAS locus for olecranon-type traits (Supplemental Table 6). The expression of 38 genes in round-type fruits and olecranon-type fruits is shown in Supplemental Tables 10, among which 8 were differentially expressed (false discovery rate (FDR) < 0.01). Eight DEGs were selected as candidates affecting olecranon-type traits, including 4 highly expressed genes in olecranon honey peach (*LOC18775282*, *LOC18772209*, *LOC18773929*, *LOC18773401*) and 4 highly expressed genes in round peach (*LOC18773079*, *LOC18773525*, *LOC18773067*, *LOC18775244*) according to the expression pattern shown in the heatmap (Fig. 6 A).

We selected 80 transcripts with 1 Mb up- or down-stream of the main GWAS locus for olecranon-type traits. Among the 80 transcripts, 39 were not expressed or showed very low expression in the round-type fruits and olecranon-type fruits and 11 transcripts (FDR < 0.01) were differentially expressed (Supplemental Table 11). According to expression pattern in the heatmap (Fig. 6B), 7 genes were highly expressed in olecranon honey peach and 4 genes were highly expressed in round peach. According to sequence comparison, *ONT.13798.5* showed no corresponding gene (Table 3). Thus, we selected 10 genes and one new gene (*ONT.13798.5*) as candidates affecting olecranon-type traits. Eight genes were identical to the candidate genes in Fig. 6 A, namely *LOC18775282*, *LOC18772209*, *LOC18773929*, *LOC18773079*, *LOC18773525*, *LOC18773067*, *LOC18775244*, *LOC18773401*, and *LOC18785015*. The other 2 genes were *LOC18772013* and *LOC18772236*. *ONT.13798.5*, a new gene, was located on scaffold_6, and its nucleic acid sequence is shown in Supplemental Table 12.

**Table 3:** Basic information on corresponding genes in the differential transcriptome

Transcript name	Gene name	Scaffold	Start	End	Gene length	Regulated	Nt_annotation
*ONT.13798.5*	--	NC_034014.1	27,013,215	27,013,728	513	down	--
*rna28213*	*LOC18775282*	NC_034014.1	26,827,826	26,829,644	1818	down	*Prunus persica* hypothetical protein (PRUPE_ppa005902mg) mRNA, complete cds
*rna28214*	*LOC18772209*	NC_034014.1	26,837,923	26,840,546	2623	down	*Prunus persica* hypothetical protein (PRUPE_ppa002340mg) mRNA, complete cds
*rna28225*	*LOC18773929*	NC_034014.1	26,914,747	26,918,463	3716	down	*Prunus persica* hypothetical protein (PRUPE_ppa007516mg) mRNA, complete cds
*rna28232*	*LOC18773079*	NC_034014.1	26,936,886	26,937,706	820	up	*Prunus persica* hypothetical protein (PRUPE_ppa013554mg) mRNA, complete cds
*rna28236*	*LOC18773525*	NC_034014.1	26,952,937	26,953,951	1014	up	*Prunus persica* hypothetical protein (PRUPE_ppa013428mg) mRNA, complete cds
*rna28237*	*LOC18773067*	NC_034014.1	26,956,281	26,958,008	1727	up	PREDICTED: *Prunus mume* RING-H2 finger protein ATL74-like (LOC103343007), mRNA
*rna28242*	*LOC18772013*	NC_034014.1	26,966,652	26,972,706	6054	down	PREDICTED: *Prunus mume* B3 domain-containing transcription repressor VAL2 (LOC103342968), transcript variant X2, mRNA
*rna28245*	*LOC18775244*	NC_034014.1	26,975,388	26,978,541	3153	up	*Prunus persica* hypothetical protein (PRUPE_ppa010719mg) mRNA, complete cds
*rna28251*	*LOC18772236*	NC_034014.1	26,987,854	26,992,354	4500	up	*Prunus persica* hypothetical protein (PRUPE_ppa000904mg) mRNA, complete cds
*rna28261*	*LOC18773401*	NC_034014.1	27,019,572	27,020,630	1058	down	PREDICTED: *Prunus mume* uncharacterized LOC103342899 (LOC103342899), mRNA

We next selected 11 DEGs that may affect olecranon-type traits, which are shown in Table 3. *ONT.13798.5* had the shortest length of 513 bp, and *LOC18772236* showed the longest length of 4500 bp (Table 3).

### Validation of expression patterns of candidate genes

**Fig. 7:**
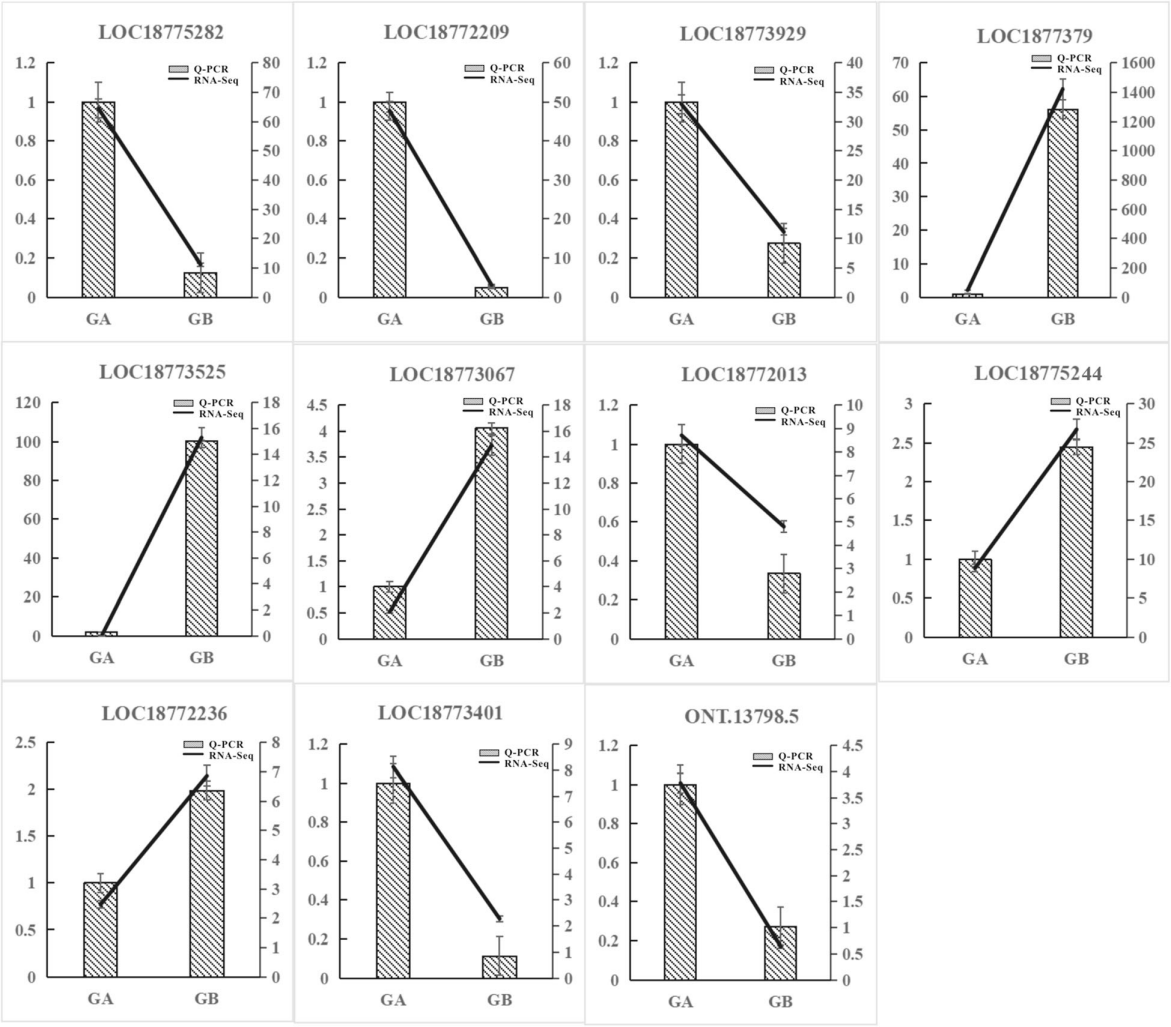
RT-PCR validation of expression levels of 11 differentially expressed genes identified by RNA-sEq. Olecranon honey peach denoted by A. Round peach denoted by B

To confirm the accuracy of the ONT full-length transcriptome sequencing results and validate the expression patterns of the candidate genes, RT-PCR of the 11 selected genes was performed (Table 3). The sequences of the gene-specific primers are shown in Table 4. The relative expression levels of all 11 genes were consistent with the results of ONT full-length transcriptome sequencing analysis (Fig. 7). Complete correlations between RNA-Seq data and RT-PCR data were also identified in most of the tested genes (R = 1). Therefore, the qRT-PCR data confirmed the reliability of the transcriptome data.

**Table 4:** Primers used for quantitative real-time RT-PCR

Gene name	Transcription ID	Forward primer (5′-3′)	Reverse primer (5′-3′)	Annealing temperature TM
*LOC18775282*	*rna28213*	GATAGCGAAGGCGGAGAA	CCCAATCATCACCACGAAAT	58
*LOC18772209*	*rna28214*	TGCTTGTACGGTACTTAA	AAATGGAGAGGTTGAGAA	58
*LOC18773929*	*rna28225*	GCTAAGAGGACCAAGAAC	CACTATCGCAAGACTGTT	58
*LOC18773079*	*rna28232*	AGTTAGTATTCCTTACAAGA	TTTATTCCGACTCTCTAAT	58
*LOC18773525*	*rna28236*	GAAGCAAGCCGTCAATGG	GCAGTCAGTAGAAGGAGAGAT	58
*LOC18773067*	*rna28237*	ACAGGACAGCAGATTCATACAT	GCACATATCAGAGCACATAACAG	58
*LOC18772013*	*rna28240*	CCATCTTACTGCCATTCACAA	CCACTTATTACAGGAAGGTTCTC	58
*LOC18775244*	*rna28245*	CACACGAATACTGAGAAA	ATGAGACATTGAAGGATG	58
*LOC18772236*	*rna28251*	AGCATCTGTTGTTGACTTG	CCTTAGTTCCTTGAGTGAGA	58
*LOC18773401*	*rna28261*	GGAGTGGTTGTTTATCTA	TTTGTTCTTGAAGTTGTG	58
*ONT.13798.5*	*ONT.13798.5*	ACTTTCAGAAGAATCAGAGATG	GTGGTGACGGCAGTTATC	58

## Discussion

Bud mutations (bud sports) are common in many plant species including fruit trees [3], and somatic genetic variation can lead to phenotypic alterations in plants [8]. There are obvious differences in the shape of round peach and olecranon honey peach (Fig. 2). Based on quality indicators, the round peach remarkably differs from olecranon honey peach, particularly in the fruit type (Table 2). These differences indicate that although these peaches are grown on a similar tree, they do not belong to the same variety. In this study, we evaluated fruit type-related indicators, including weight, horizontal and vertical diameters, fruit type index, and the part of the fruit that resembles an eagle’s beak. The bud mutant variant of round peach was typically smaller than that of olecranon honey peach based on these indicators. The olecranon honey peach is a new variety with high consumer value in China [4]. However, not all bud sports have a regulatory effect on the fruit type, such as the Beni Shogun apple mutation. This mutation reportedly affects traits such as skin coloration, fruit softening, and starch hydrolysis, whereas the loss of acidity, sugar accumulation, and weight appear to be unaffected by the mutation [9].

GWAS can be used to identify the genes or quantitative trait loci underlying complex traits, such as in melon [5], cotton [10], rapeseed [11], grape [12], and peach [5, 7, 13]. In previous studies of peach fruit shape, some candidate genes for forming the flat peach have been identified, such as *ppa003772m* [5] and *ppa025511m* [6], but olecranon-type traits have not been evaluated. In the current study, the olecranon-type trait was located close to the candidate genes identified by linkage analysis. *ppa003772m* and *ppa025511m* were not included within 1 Mb up- or down-stream of the main GWAS locus for olecranon-type traits (Supplemental Table 6). Thus, these genes related to eagle beak traits are not related to *ppa003772m* and *ppa025511m*. However, a recently reported candidate gene (*PpCAD1*) for flat-shaped peach at 26,924,482 bp (Supplemental Table 6) is annotated by constitutively activated cell death in GENES1 [5]. *PpCAD1* did not differ in the round peach and olecranon honey peach according to ONT full-length sequencing.

Furthermore, to better understand the formation of olecranon-type traits in peach at the molecular level, combined ONT full-length sequencing was performed to comprehensively analyze the transcriptome profile in the olecranon honey peach and round peach. This method overcomes technical limitations by accurately predicting full-length splicing subtypes in second-generation sequencing short-read data [14]. ONT full-length sequencing technology has been used to sequence plants and animals, such as polar bear [15], *Hiptage benghalensis* [16], *zebrafish* [17], and *Susscrofa* [18]. Compared with previous sequencing results [19, 20], the ONT full-length sequencing produced not only longer reads, but also a higher comparison ratio of reads.

Fruit ripening is a developmental process, and fruit shape depends on events that occur early in fruit development [15, 21, 22]. In flat peach, as the typical peach evaluated in fruit shape research, 28 genes related to fruit shape have been identified [2]. Based on their functional annotations and coding sequences, 3 genes were identical among the 11 identified candidate genes, indicating similar mechanisms in regulation of the olecranon-type trait and flat-type trait. Two of three genes are annotated as non-specific lipid-transfer protein (*LOC18773079*, *LOC18773525*) and the other is annotated as leucine-rich repeat receptor-like protein (*LOC18772236*). As shown in Fig. 7, high expression of *LOC18773079*, *LOC18773525*, and *LOC18772236* inhibits the formation of olecranon. In tomato, four fruit shape genes have been identified [22], named as *SUN*, *OVATE*, *FAS*, and *LC*. In eggplant, *SUN* and *OVATE* exert conserved functions in controlling the fruit shape [23, 24]. *FAS* expression leads to a flat or oxheart shape, and *LC* expression controls the meristem size and locule number. No genes belonged to the *FAS* and *LC* types among the 11 candidate genes. *SUN* and *OVATE* are related to fruit elongation. According to GO annotation, *LOC18775244* is related to translational elongation, and its low expression is favorable for the formation of olecranon (Fig. 7). These results contrast those determined by Jian [2] for the flat-type peach, who found that the mechanisms regulating fruit shape between tomato and peach are similar, and those regulating the shape of olecranon and flat peach may differ.

In addition, according to NR annotation, *LOC18775282* is related to CBL-interacting protein kinases and highly expressed in olecranon peach. In a study of cotton fiber elongation [25], CBL-interacting protein kinases were also linked to fiber length. *LOC18772013* is related to B3 domain-containing protein. As a member of the B3 DNA binding superfamily, auxin-responsive factors can bind to auxin-responsive elements of induced genes and further regulate plant metabolism [26]. Auxin plays an important role in plant development at the cellular level, such as cell extension, division, and differentiation [27]. *LOC18773067* was annotated as RING-H2 finger protein ATL74. RING-H2 finger protein plays key roles in regulating growth/developmental processes, hormone signaling, and responses to biotic and abiotic stresses in plants [28]. U-box domain-containing protein and serine/threonine-protein also involved in olecranon formation.

## Conclusions

The shape of fruit is an important characteristic affecting consumer decisions. The shape of the top of olecranon honey peach fruit resembles the beak of an eagle, which is popular among consumers. No studies have been performed to examine the olecranon-type trait. In this study, loci associated with the olecranon-type trait were examined by GWAS. The gene loci related to the olecranon-type trait was 26,924,482 bp in NC_034014.1. To further identify olecranon-type trait genes, we performed ONT full-length transcriptome sequencing of young fruits of 3 olecranon honey peaches and 3 round peaches. The sequencing data of each sample reached up to 7.10GB, with 14,360 genes and 23,167 transcripts expressed in both olecranon and round peach. Eleven DEGs were selected as candidate genes, six of which were highly expressed in olecranon peach and named as *LOC18775282*, *LOC18772209*, *LOC18773929*, *LOC18772013*, *LOC18773401*, and *ONT.13798.5*. Five of these were highly expressed in round peach and named as *LOC18773079*, *LOC18773525*, *LOC18773067*, *LOC18775244*, and *LOC18772236*. The genes were within 1 Mb up- or down-stream of the main GWAS locus for olecranon-type traits. Notably, *ONT.13798.5* is a new gene, and *LOC18775244* is related to translational elongation. The formation of olecranon traits is a complex process, which has been shown to be related to cellular signal transduction and plant hormones. These results provide a genetic basis for the olecranon-type trait and can be used to further evaluate the eagle beak shape of olecranon honey peach.

## Methods

### Plant materials and sample collection

A total of 456 peach accessions were collected in China for GWAS and are distributed in the North (315), Northwest (26), Qinghai-Tibet Region (3), South (83), Yangtze River Basin (22), and Yunnan-Guizhou Plateau (7) regions in China (Fig. 1 A). Detailed information on these samples is shown in Supplementary Table 1. The collected peach leaves were immediately frozen at − 80 ℃ for DNA extraction in liquid nitrogen.

In addition, bud mutation was found on a tree of olecranon honey peach in Lianping in northern Guangdong province, China, which was used for full-length transcriptome studies. One branch of this tree produces normal round-shaped fruit, whereas other branches produce olecranon-shaped fruit (Fig. 2). The tree was maintained in the same manner as other peach trees in the orchard. The bud sport was stable for at least 3 years. The round-type fruit was obtained by selecting the branch with the bud sport. To avoiding sampling of chimeras, we selected olecranon honey peaches produced far from the mutated branch. After the olecranon traits had formed, the tops of young fruits (young fruit development) were immediately frozen in liquid nitrogen and stored at − 80 ℃ for full-length transcriptome sequencing and RT-PCR.

Ripening fruits were selected by experienced growers (fruit hanging time of approximately 75 days) and quickly transported to a well-ventilated, sterile laboratory to count the olecranon type traits. The olecranon trait was defined as the tip of a peach shaped like an eagle’s beak.

### DNA extraction and sequencing

Genomic DNA was extracted from fresh young leaves using the cetyltri*methyl*ammnonium bromide method [29]. At least 5 µg of genomic DNA was used for each accession to construct sequencing libraries according to the manufacturer’s instructions (Illumina, San Diego, CA, USA). The libraries were sequenced on an Illumina HiSeq X Ten or NovaSeq 6000 platform, generating 150-bp paired-end reads. Raw sequencing reads were trimmed using Btrim software to filter out low-quality bases and sequences using the following parameters: remove adapter reads; pair-end reads with > 10 % “N” bases; average base-quality of less than 10; quality score of 3′ bases < 50.

### SNP and InDel calling

High-quality reads were compared with the peach “NCBIv2” genome using Burrows-Wheeler Aligner (version 0.7.10-r789) with the following parameters: bwa mem 4 –k 19 -M. Next, the alignment bam/sam files were subjected to a series of processing and filtering steps, including position sorting, repeated read marks and removal of local realignments, and basic quality recalibration using Picard (https://sourceforge.net/projects/picard/) and GATK software (v3.8) [30]. SNPs and InDels were filtered by using the HaplotyPecalller tool in GATK software package. SNPs and InDels were annotated based on the *Prunus persica* (assembly Prunus_persica_NCBIv2) genome using snpEff software [31], and SNPs were categorized into intergenic regions, upstream or downstream regions, and exons or introns.

### LD analysis and GWAS

LD was calculated between each pair of SNPs on the same chromosome using PLINK2 software [32]. The values of the squared correlation coefficient (r^2^) and significance of any LD detected between polymorphic sites were analyzed in all chromosomes with a 1000-kb window. LD blocks were defined based on the following parameters [33]: minor allele frequency > 0.05, r^2^, ldwindow 999,999, ld-window- r2 0. GWAS of the leaves was based on high-confidence SNPs (minor allele frequency > 0.05, INT > 0.8) from 456 accessions. The Q-value was calculated with ADMIXTURE [34] software, which represents the population structure. The K-value was calculated using SPAGeDi [35] software, which represents the genetic relationship between samples. FaST-LMM [36] was used to test the associations between samples. Both the Q- and K-value were used in TASSEL. The P-value of each SNP was calculated, and –log10P > 7 was defined as the suggestive threshold and genome-wide control threshold.

### RNA extraction and transcriptome sequencing

RNA was extracted from six peach samples representing three biological replicates of two peach cultivars. RNA extraction was performed using TRIZOL reagent (Invitrogen, Carlsbad, CA, USA) according to the manufacturer’s instructions. RNA concentration and purity were analyzed with a Nanodrop ND-1000 spectrophotometer (NanoDrop Technologies, Wilmington, DE, USA) and an Agilent 2100 Bioanalyzer (Agilent Technologies, Santa Clara, CA, USA). Three biological replicates were evaluated for each sample. For ONT RNA sequencing, individual cDNA libraries were constructed from total RNA samples with the SQK-LSK109 RNA Library Prep Kit for Oxford Nanopore (ONT) according to the manufacturer’s instruction. The cDNA libraries were sequenced using a PromethlON sequencer (ONT). Primer annealing, reversing transcription into cDNA and plus switch oligo, synthesis of complementary strands, DNA damage and end repair, and magnetic bead purification were performed. Finally, a sequencing linker was added.

### Analysis of ONT sequencing data

Raw ONT sequencing reads were filtered to remove low-quality reads (Qscore ≤ 7 and minimum read length ≤ 500 bp). Full-length non-chimeric transcripts were obtained. Clusters of full-length non-chimeric transcripts were obtained after mapping to the reference genome with mimimap2, and consensus isoforms were obtained by polishing within each cluster by pinfish. Redundant consensus sequences were removed by mapping the consensus sequences to the reference genome using minimap2 [37]. Mapped reads were further collapsed by using cDNA Cupcake package with coverage ≤ 0.85, identity ≤ 0.90, and merge sequences showing differences only in the 5′ exons.

For comprehensive functional annotation, the full-length transcripts were analyzed by BLAST against public protein databases, including the NR (NCBI non-redundant protein sequences) database, Pfam (Protein family) database, KEGG database [38], KOG/COG of proteins database [39, 40], Swiss-Prot database [41], and GO database [42].

### Quantification of gene/transcript expression levels and differential expression analysis

Full-length reads were mapped to the reference transcriptome sequence. Reads with a match quality > 5 were further quantified. Expression levels were estimated by reads per gene/transcript per 10,000 mapped reads. Prior to differential gene expression analysis, for each sequenced library, the read counts were adjusted using the edgeR program package with the one scaling-normalized factor. Differential expression analysis of two samples was performed using the EBSeq R package. The resulting FDR was adjusted using the posterior probability of being differentially expressed. FDR < 0.01 and fold-change ≥ 2 were used as thresholds for significantly different expression. The identified DEGs and DETs were analyzed for GO and KEGG enrichment.

### Validation of gene by quantitative real-time RT-PCR

RNAs were isolated from the top of the olecranon honey peach and round peach. Two biological replicates were included. For quantitative real-time RT-PCR analysis, reverse transcription was performed using the TUREscript 1st Stand cDNA SYNTHESIS Kit with cDNA Eraser (Aidlab, Beijing, China) according to the manufacturer’s instructions. Gene- or transcript-specific primers were designed with Primer Express 3.0 software (Applied Biosystems, Foster City, CA, USA). qPCR was performed on an Applied Biosystems 7500 RealTime PCR System using 2×SYBR® Green qPCR Master Mix (Qiagen, Hilden, Germany). *LOC18778082* was selected as a reference gene. The relative expression level was calculated using the following formula: fold-change = 2^−△△CT^.

### Statistical analyses

Data are expressed as the mean ± standard deviation of three replicates. Significant differences between the means of different parameters were calculated by Duncan’s multiple-range test using SPSS 17.0 software (SPSS, Inc., Chicago, IL, USA). *P* < 0.05 was considered to indicate statistically significant results.

## Supplementary Information

**Additional file 1: Supplemental Table S1.** List of 456 peach accessions analyzed in this study. The code, name, scientific name, area, and plantation for 456 re-sequenced peach accessions. Olecranon indicates whether the olecranon-type trait is present; 1 means yes, 0 means no, NA means no statistics. **Supplemental Table S2.** Sequence information on the genomes of 456 peach accessions. N (%) indicates the percentage of failed sequencing nucleotides. Q30 (%) indicates the percentage of 99.9% well-sequenced nucleotides. Ave_depth indicates average covering depth of sample. Cov_ratio_*(%) indicates the percentage of bases at or above a given depth of coverage as a percentage of the total base number of the reference genome. Properly mapped (%) indicates the percentage of the sequenced data mapped to the reference genomes. **Supplemental Table S3.** Single-nucleotide polymorphism statistics detected between sample and reference genome. **Supplemental Table S4.** Statistics of single-nucleotide polymorphism annotation results of samples **Supplemental Table S5.** Statistics of insertion-deletion annotation results of samples. **Supplemental Table S6.** Statistical table of genes within 1Mb upstream and downstream of main genome-wide association study sites of olecranon type traits. **Supplementary table S7.** Clean data statistical table. After the original fastq data were further filtered for short reads and low-quality reads, the total clean data were obtained. A-1; A-2; A-3: Olecranon honey peach; B-1; B-2; B-3: Round peach. **Supplementary table S8.** Full-length sequence and comparison with reference transcriptome results. **Supplementary table S9.** Statistical table of the number of annotated genes and transcripts. **Supplementary table S10.** Statistics of gene expression in round peach and olecranon peach.**Supplementary table S11.** Statistics of transcripts expression in the round peach and the olecranon peach. The nucleic acid sequence of the ONT gene is as follows.

**Additional file 2: Supplemental Figure S1.** Heat map of expression correlation between two samples. Clean data statistical table. After the original fastq data were further filtered for short reads and low-quality reads, the total clean data were obtained. A-1; A-2; A-3: Olecranon honey peach; B-1; B-2; B-3: Round peach.

**Additional file 3: Supplemental Figure S2.** KEGG classification map of differentially expressed genes. The ordinate is the name of the KEGG metabolic pathway and abscess is the number of genes annotated to the pathway and its proportion to the total number of genes annotated.

**Additional file 4: Supplemental Figure S3.** KEGG classification map of differentially expressed transcripts.

## Data Availability

The original datasets analyzed in the current study are available in the NCBI Sequence Read Archive (SRA). The date of Olecranon peach Nanopore transcriptome sequencing link to obtain data is https://www.ncbi.nlm.nih.gov/bioproject/PRJNA754788. The date of GWAS link to obtain data is https://www.ncbi.nlm.nih.gov/bioproject/PRJNA758752 .

## Abbreviations

GWAS: genome-wide association study.
NR: NCBI non-redundant protein sequences.
Pfam: Protein family.
KEGG: Kyoto Encyclopedia of Genes and Genomes.
COG: Cluster of Orthologous Groups.
GO: Gene Ontology.
DEGs/DETs: Differentially expressed genes/transcripts.
