# Supplementary figures and images for: Genome-wide association study and transcriptome of olecranon-type traits in peach (Prunus persica L.) germplasm
Source: BMC Genomics. 2021 Sep 28;22:702. doi: 10.1186/s12864-021-08017-y (PMC8480057; doi:10.1186/s12864-021-08017-y)

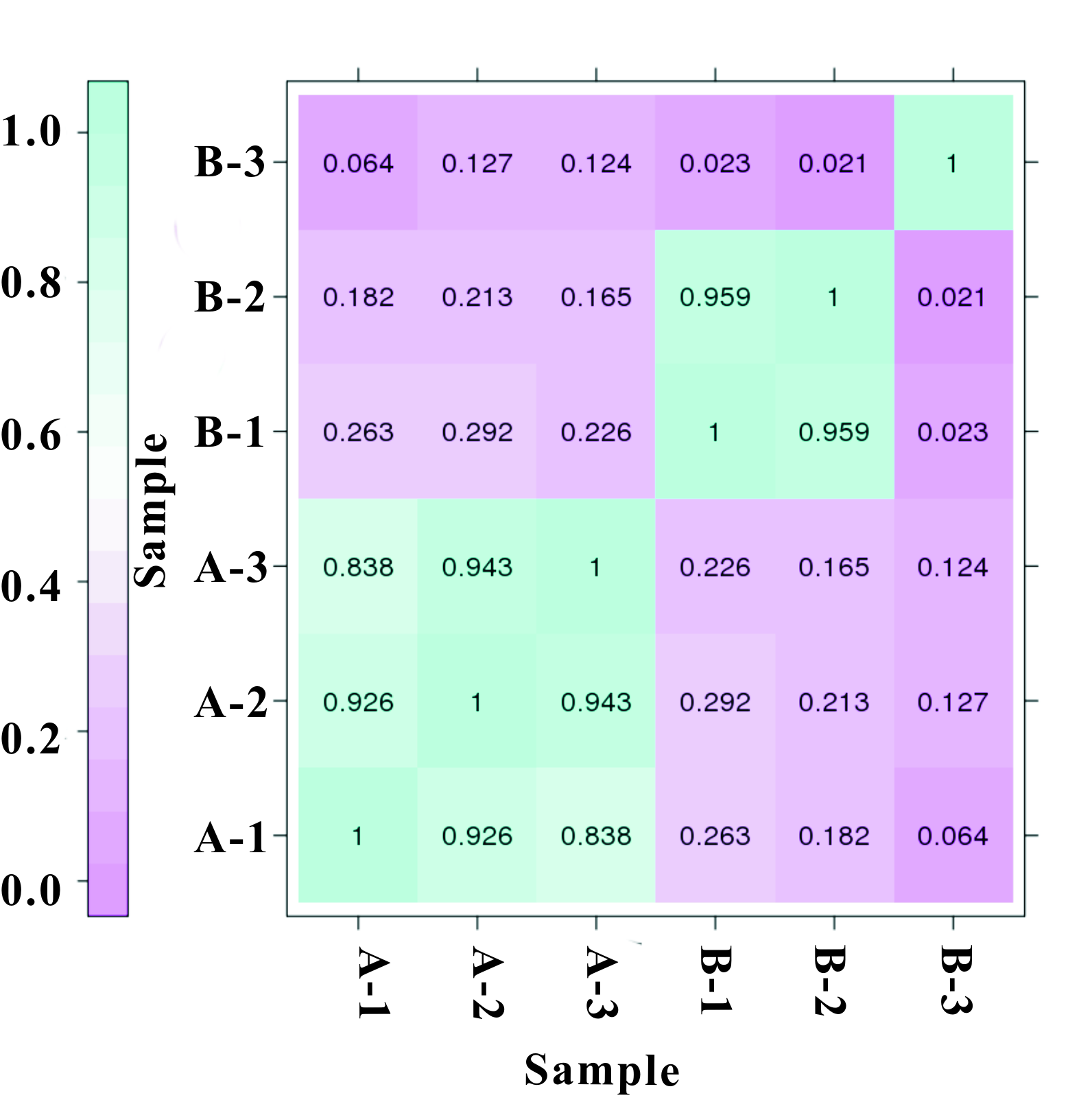

Supplement: Supplementary file 2 — Additional file 2: Supplemental Figure S1. Heat map of expression correlation between two samples. Clean data statistical table. After the original fastq data were further filtered for short reads and low-quality reads, the total clean data were obtained. A-1; A-2; A-3: Olecranon honey peach; B-1; B-2; B-3: Round peach. [file 12864_2021_8017_MOESM2_ESM.tif]

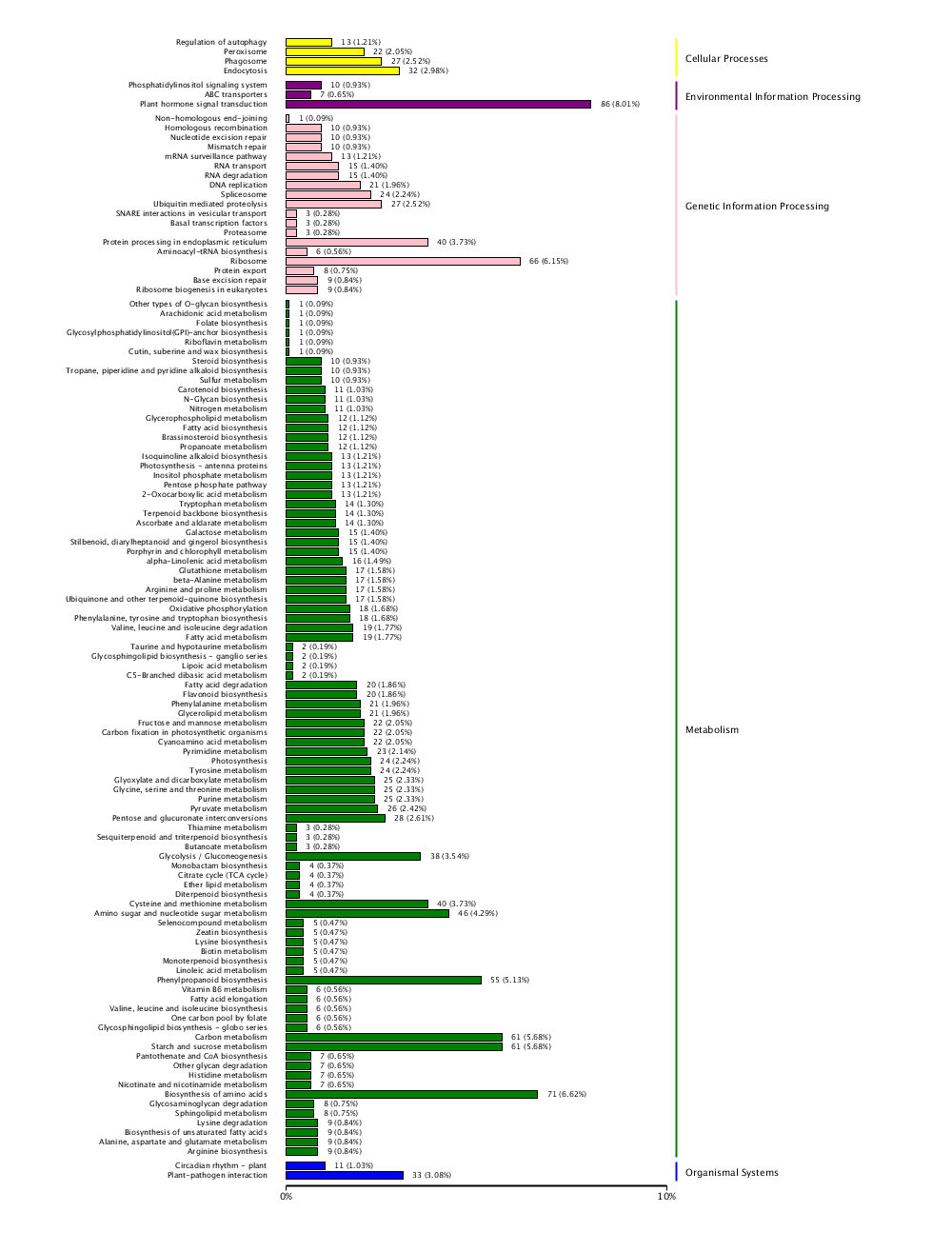

Supplement: Supplementary file 3 — Additional file 3: Supplemental Figure S2. KEGG classification map of differentially expressed genes. The ordinate is the name of the KEGG metabolic pathway and abscess is the number of genes annotated to the pathway and its proportion to the total number of genes annotated. [file 12864_2021_8017_MOESM3_ESM.png]

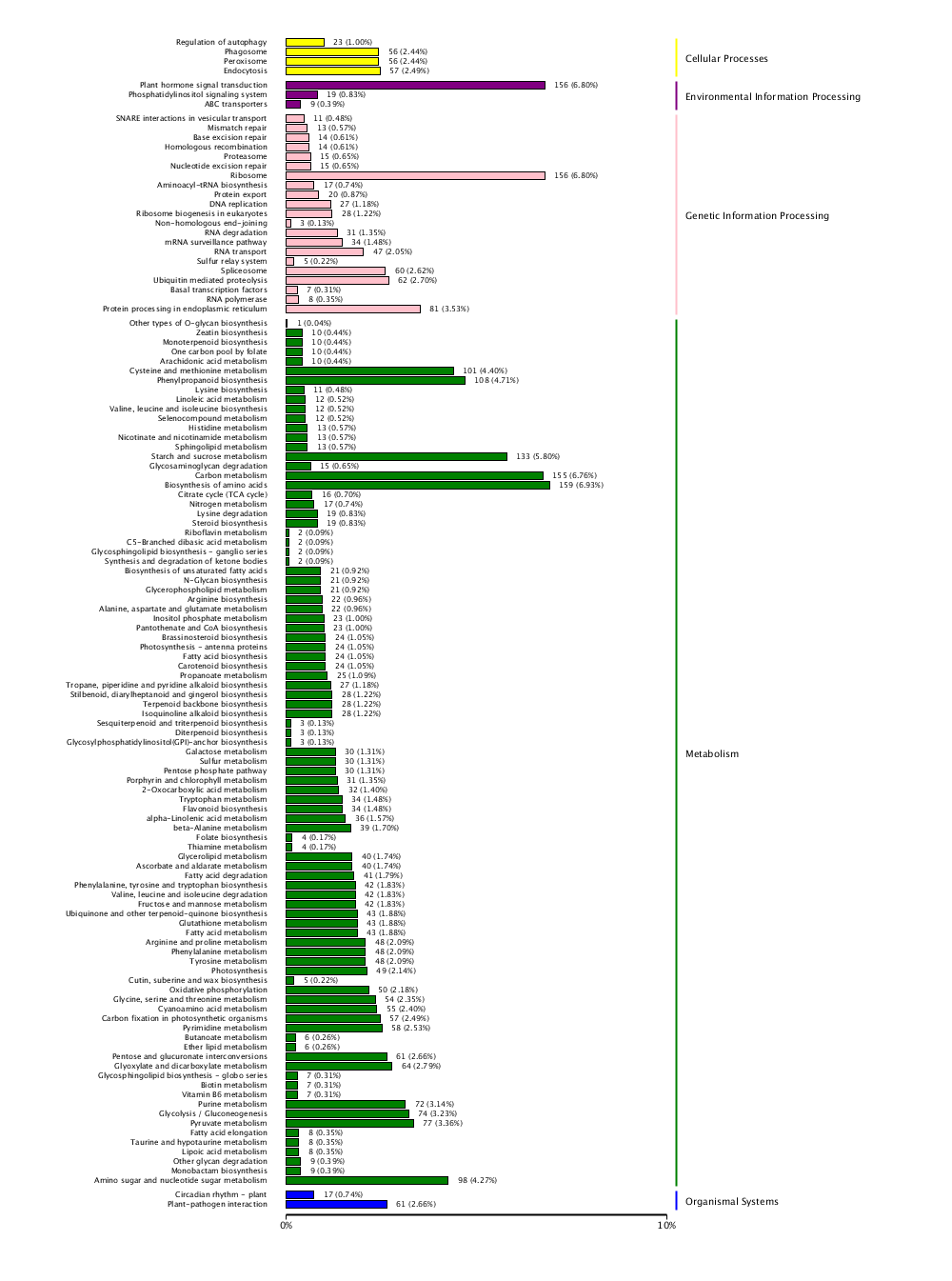

Supplement: Supplementary file 4 — Additional file 4: Supplemental Figure S3. KEGG classification map of differentially expressed transcripts. [file 12864_2021_8017_MOESM4_ESM.png]
